# Assessing the repurposing potential of disease-modifying antirheumatic drug targets to reduce Alzheimer's disease risk: a Mendelian randomization study

**DOI:** 10.1016/j.bbih.2026.101185

**Published:** 2026-01-30

**Authors:** Christina N. Kushnir, Victoria Taylor-Bateman, Neil M. Davies, Emma L. Anderson

**Affiliations:** a Division of Psychiatry, University College London, London, WC1E 6BT, UK; b Department of Statistical Science, University College London, London, WC1E 6BT, UK; c Department of Public Health and Nursing, Norwegian University of Science and Technology, Norway

**Keywords:** Drug target Mendelian randomization, DMARD, Inflammation, Alzheimer's disease, Dementia

## Abstract

**Background:**

Systemic inflammation plays a key role in the development and progression of Alzheimer's disease (AD). However, the repurposing potential of select anti-inflammatory drug targets for AD treatment remains unclear.

**Methods:**

Two-sample Mendelian randomization (MR) and colocalization analyses were conducted to estimate the effects of select disease-modifying antirheumatic (DMARD) targets on AD risk. We investigated 9 DMARD targets, using blood protein quantitative trait loci (pQTLs) from the UK Biobank Pharma Proteomics Project (*n* = 54,219). Outcome associations were extracted from the International Genomics of Alzheimer's Project (*n*cases = 21,982, *n*controls = 41,944).

**Results:**

Our MR estimates suggest that higher levels of *FCGR3B*, an etanercept target, increased the risk of AD (OR: 1.10; 95% CI [1.02, 1.19]; *p* = 0.01). We found little evidence that the remaining DMARD targets affected AD risk. Colocalization analysis provided little evidence that target pQTLs, including *FCGR3B*, colocalized with AD.

**Conclusions:**

Our findings suggest a causal effect of *FCGR3B* on AD risk, but not for the remainder of the analyzed DMARD targets. Further research is recommended to elucidate the causal role of *FCGR3B* in AD and build upon the current literature on viable AD therapeutic targets.

## Highlights

• This is the first MR and colocalization study on select DMARD targets and AD risk, using *cis*-pQTLs as instruments.
• MR suggests that higher plasma *FCGR3B* levels increase the risk of AD.
• There was little evidence of an effect for the remaining targets.
• This suggests that the majority of these targets in plasma are unlikely to be effective candidates for reducing AD risk.

## 1 Introduction

Globally, an estimated 57 million people currently suffer from dementia, with this number expected to increase nearly threefold by the mid-21st century (Nichols et al., 2022). Alzheimer's disease (AD), the most common cause of dementia and a leading cause of death in the United States and the United Kingdom, imposes both immense pain on patients and their loved ones as well as a significant socioeconomic burden on healthcare systems, making it imperative to find treatment candidates that do not simply address quality of life but, more urgently, target pathological mechanisms that may prevent or halt disease progression (National Center and for Health Statistics, 2024; Nandi et al., 2024).

In recent years, considerable evidence has implicated chronic and systemic inflammation in the pathology of AD (Bayraktaroglu et al., 2025; Kinney et al., 2018). Prolonged microglial activation resulting from the presence of harmful agents, e.g., in the context of AD, amyloid β (Aβ) deposits, triggers the excessive release of pro-inflammatory cytokines, leading to further neuronal toxicity and tissue damage (Kinney et al., 2018; Swardfager et al., 2010). Subsequent deregulation of key pro-inflammatory mediators, such as tumor necrosis factor (TNF-α) and interleukin-6 (IL-6), has been linked to additional hallmark molecular mechanisms contributing to AD progression (Kinney et al., 2018; Swardfager et al., 2010; Jayaraman et al., 2021; Erta et al., 2012; Chang et al., 2017). Several genome-wide association studies (GWAS) have substantiated the role of immune function and inflammation in AD, with the largest and most recent AD GWAS to date identifying multiple genome-wide significant loci near genes involved in innate immunity and microglial function, including triggering receptor expressed on myeloid cells 2 (*TREM2*), phospholipase C-γ2 (*PLCG2*), and progranulin (*GRN*) (Bellenguez et al., 2022). This information taken together provides converging evidence that dysregulated immune responses via chronic inflammation contribute to AD pathogenesis, highlighting inflammatory pathways as promising therapeutic targets.

Disease-modifying antirheumatic drugs (DMARDs), a class of anti-inflammatory medications that modulate cytokine activity and other pro-inflammatory targets, may be potentially viable drug repurposing candidates for AD prevention and treatment. Importantly, the disease-modifying component of DMARDs makes them encouraging therapeutic candidates to counter the progressive neurodegeneration that characterizes AD. However, observational studies examining the association between prescribed DMARD usage and the risk of AD have yielded mixed and contradictory results (Chou et al., 2016; Huang et al., 2019; Xie et al., 2024; Sattui et al., 2022; Watad et al., 2022; Zhou et al., 2020). Furthermore, such studies investigate individuals already affected by autoimmune and/or rheumatic diseases, making confounding by indication highly likely. While randomized control trials (RCTs) may help clarify the existing research, only two RCTs have investigated the casual effects of DMARDs on the risk of AD, both with small sample sizes (*n* = 41, *n* = 15) (Butchart et al., 2015; Tobinick et al., 2006). These RCTs examined the use of etanercept, a commonly prescribed DMARD, over the course of 6 months with results from both trials suggesting a protective effect on AD risk. However, the sample size and lack of statistical power limits inference from these studies.

**Fig. 1:**
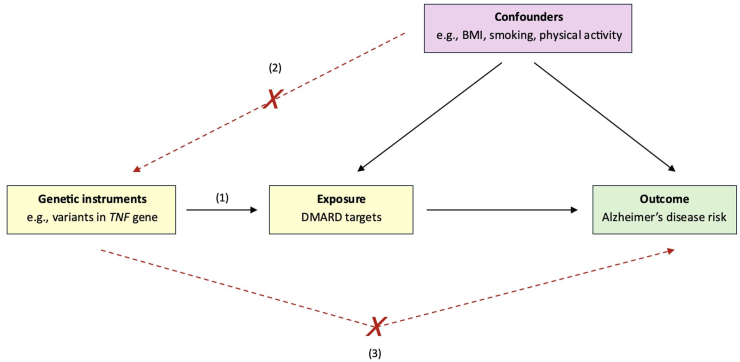
Diagrammatic illustration of the study. MR assumes that the genetic variant (1) is robustly associated with the exposure variable (relevance); (2) has no shared confounders with the outcome (independence); and (3) affects the outcome only through the exposure (exclusion restriction) (Davies et al., 2018). Abbreviations: DMARD, disease-modifying antirheumatic drug; *TNF*, tumor necrosis factor; BMI, body mass index.

Mendelian randomization (MR) is a statistical approach that uses genetic variants as instrument variables to estimate the casual effect of the exposure on a disease outcome (Smith and Ebrahim, 2004). Due to the random inheritance of genotypes at conception, genetic associations with a risk factor or disease outcome are less likely to be affected by confounders and reverse causality, which are pervasive in observational studies, therefore aiding causal inference (Smith and Ebrahim, 2004). Additionally, MR can be employed to study the effects of perturbing drug targets by instrumenting variants associated with circulating protein levels or gene expression of the drug target protein (Fig. 1) (Gill et al., 2021). Drug target MR follows the rationale that the instrumented variants mimic or “proxy” the perturbation of these proteins by drugs known to target these mechanisms and can thus allow for the exploration of drug effects on a disease outcome (Gill et al., 2021). Colocalization often accompanies MR studies as a form of sensitivity analysis to reduce the threat that linkage disequilibrium (LD) poses to the assumptions of MR. As neither MR nor colocalization alone is sufficient to establish causality, the integration of both methods can strengthen inferences in statistical genetic studies. Here, we assess whether select DMARD targets are likely to be efficacious therapeutic targets for reducing AD risk, using two-sample MR and colocalization.

## 2 Methods

### 2.1 Study design

**Fig. 2:**
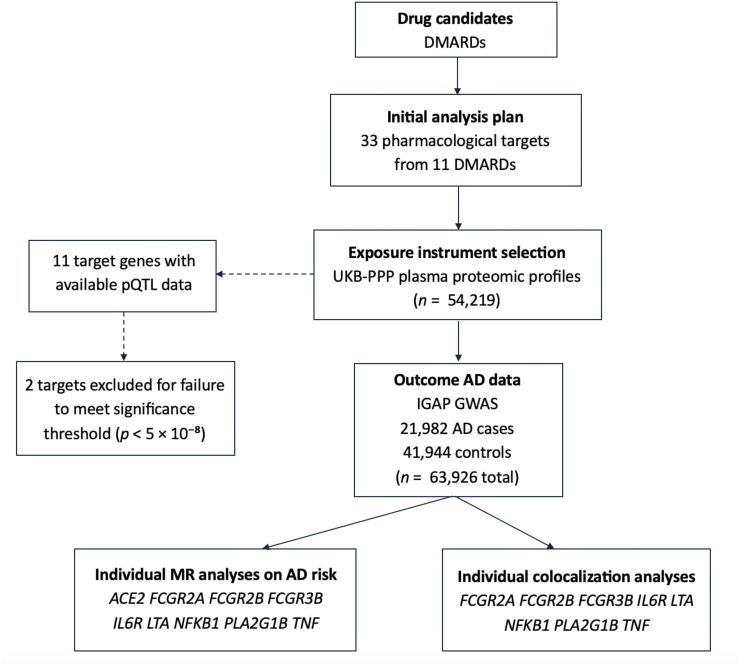
Study design. Abbreviations: DMARDs, disease-modifying antirheumatic drugs; UKB-PPP, UK Biobank Pharma Proteomics Project; pQTL, protein quantitative trait loci; AD, Alzheimer's disease; IGAP, International Genomics of Alzheimer's Project; GWAS, genome-wide association study; MR, Mendelian randomization.

A flow diagram of the study design is shown below (Fig. 2). We instrumented protein quantitative trait loci (pQTLs), defined as variants in the genome associated with circulating protein levels, to genetically proxy the effects of perturbed DMARD targets. We performed MR on disease outcomes and then followed with colocalization analyzes between target pQTLs and AD in the *cis*-regions of target-encoding genes.

### 2.2 Selection of DMARD targets

11 DMARDs, totaling 33 target genes, were initially selected for analysis. The rationale for inclusion was based on findings from the current literature regarding associations between DMARD use and dementia or AD incidence (Chou et al., 2016; Huang et al., 2019; Xie et al., 2024; Sattui et al., 2022; Watad et al., 2022; Zhou et al., 2020). The drugs identified were methotrexate, sulfasalazine, hydroxychloroquine, leflunomide, adalimumab, etanercept, infliximab, certolizumab, golimumab, tocilizumab, and tofacitinib. Methotrexate and tofacitinib had no available pQTL data and were therefore excluded from analysis. In later stages, the pQTL data for the leflunomide target, *DHODH*, did not meet the required significance threshold; as a result, leflunomide was also excluded from analysis. DMARDs and their corresponding encoding gene targets were identified using the DrugBank database (https://go.drugbank.com) and are listed in Table 1. Note that this table does not encompass all targets of the respective DMARDs, but rather only those with available pQTL data. Additionally, some targets, such as *TNF*, are shared across multiple drugs in our analysis.

**Table 1:** Selected DMARDs and their analyzed targets.

Drug name	Drug target
Sulfasalazine	*PLA2G1B*
	*NFKB1*
Hydroxychloroquine	*ACE2*
Etanercept	*LTA*
	*FCGR2A*
	*FCGR2B*
	*FCGR3B*
	*TNF*
Adalimumab	*TNF*
Infliximab	*TNF*
Certolizumab	*TNF*
Golimumab	*TNF*
Tocilizumab	*IL6R*

### 2.3 pQTL instrument selection

Exposure data for selected DMARD targets were sourced from a large-scale proteogenomic analysis as part of the UK Biobank Pharma Proteomics Project (UKB-PPP, *n* = 54,219), which provides detailed mapping of 2923 proteins and their associations with genetic variants within blood plasma (Sun et al., 2023). We searched for *cis*-acting single nucleotide polymorphisms (SNPs) known to be associated with circulating protein levels of encoded DMARD targets (Fig. 2). Lead pQTLs were identified to proxy the effects of select DMARD target perturbations, based on independence and strength of association (meeting the genome-wide significance threshold of *p* < 5 × 10^−8^) (Risch and Merikangas, 1996). The following selection criteria were applied for instrumentation: 1) pQTLs within ±1000 kb of the gene region (*cis*-acting); and 2) a genome-wide significance threshold of *p* < 5 × 10^−8^.

### 2.4 Outcome data

Effect estimates of instrumented pQTLs were extracted from a large genome-wide association meta-analysis conducted by Kunkle et al. (2019) as part of the International Genomics of Alzheimer's Project (IGAP) (Kunkle et al., 2019). Stage 1 of the GWAS included 21,982 cases and 41,944 controls, all of European ancestry. Risk of late-onset AD (LOAD) was defined as symptom onset at ≥ 65 years of age (Kunkle et al., 2019; Querfurth and LaFerla, 2010). Diagnostic assessment occurred either in clinical evaluation or postmortem autopsy. The mean age at onset for AD cases ranged from 71.1 to 82.6 years, and the mean age at examination (or last follow-up) for healthy controls ranged from 51.0 to 78.9 years. Further information on quality control and methodology can be found in the study reference (Kunkle et al., 2019).

### 2.5 Mendelian randomization

We performed MR to estimate the individual effects of each drug target on the risk of AD. Due to the outcome GWAS solely containing autosomal data, we did not include SNPs located within the X chromosome during analysis. PQTLs were clumped using a threshold of *r*^2^ = 0.001 and a 10,000 kb window to ensure independent variants (Hemani et al.). The exposure data was coded using the most recent genome reference consortium human build 38 (GRCh38), assembly Hg20, while the IGAP GWAS outcome data used GRCh37 with assembly Hg19. Thus, any pQTLs not found in the outcome dataset were replaced by proxy variants in high LD from the 1000 Genomes European reference panel (*r*^2^ > 0.8). During data harmonization, palindromic SNPs were excluded if the minor allele frequency was >0.4 (Hartwig et al., 2016). Our primary analysis used the inverse variance weighted (IVW) estimator. We present odds ratios (ORs) and 95% confidence intervals (CIs) for AD outcomes per 1 standard deviation (SD) increase in blood plasma protein concentration.

### 2.6 Colocalization analysis

Colocalization was performed on *cis*-regions of the encoding target genes of interest (±500 kb window) to assess if identified associations with AD resulted from shared causal variants. *ACE2* was excluded from this portion of the analysis as it is located on the X chromosome (Xp22.2) and could not be matched with the outcome data. The following priors were set: SNPs within the default window and exclusively associated with trait 1 had a probability of *p*_1_ = 10^−4^; SNPs within the default window and exclusively associated with trait 2 had a probability of *p*_2_ = 10^−4^; and SNPs within the default window and associated with both traits 1 and 2 had a probability of *p*_12_ = 10^−5^ (Giambartolomei et al., 2018). A posterior probability for shared causal variants (PP.H4 ≥ 80%) was considered to demonstrate strong evidence of colocalization. A threshold of PP.H4 ≥ 50% was considered suggestive evidence.

## 3 Results

### 3.1 Effects of DMARD targets on the risk of AD

**Fig. 3:**
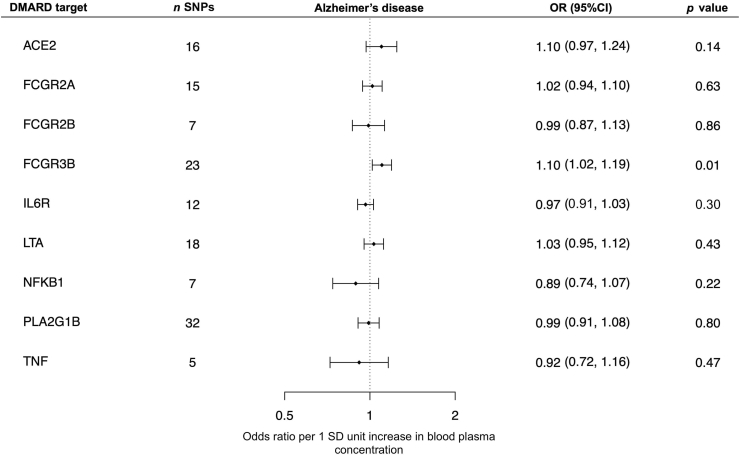
Estimates of the effects of selected DMARD targets on the risk of AD. Error bars correspond to the 95% confidence intervals. Abbreviations: DMARD, disease-modifying antirheumatic drug; SNP, single nucleotide polymorphism; OR, odds ratio; SD, standard deviation.

Overall, there was little evidence in our study that DMARD targets affected the risk of AD (Fig. 3). There was little evidence that the sulfasalazine targets, *PLA2G1B* (OR 0.99; 95% CI [0.91, 1.08]; *p* = 0.80) and *NFKB1* (OR 0.89; 95% CI [0.74, 1.07]; *p* = 0.22), affected AD risk. There was little evidence for the hydroxychloroquine target, *ACE2* (OR 1.10; 95% CI [0.97, 1.24]; *p* = 0.14). There was additionally little evidence for the targets of tocilizumab, the TNF inhibitors, and etanercept (excluding *FCGR3B*), including *LTA* (OR 1.03; 95% CI [0.95, 1.12]; *p* = 0.43), *FCGR2A* (OR 1.02; 95% CI [0.94, 1.10]; *p* = 0.63), *FCGR2B* (OR 0.99; 95% CI [0.87, 1.13]; *p* = 0.86), *TNF* (OR 0.92; 95% CI [0.72, 1.16]; *p* = 0.47), and *IL6R* (OR 0.97; 95% CI [0.91, 1.03]; *p* = 0.30). However, we observed that increased plasma levels of *FCGR3B*, an etanercept target, were associated with an increased risk of AD (OR 1.10; 95% CI [1.02, 1.19]; *p* = 0.01).

### 3.2 Results of colocalization analysis

Colocalization analyses were consistent with the MR analyses above. There was little evidence of colocalization between any of the targets and AD (PP.H4 < 10% across all proteins). Although there was evidence of a causal effect in the main MR analysis, *FCGR3B* and AD did not colocalize in the flanked chromosome 1 region of *FCGR3B* (PP.H4 = 6.21%). Colocalization results for the remainder of the targets, excluding *ACE2*, can be found in Table 2.

**Table 2:** Genetic colocalization analysis between DMARD targets and AD.

Exposure	No associations	Exposure only	Outcome only	Distinct causal variants in LD	Shared causal variant
	PP.H0.abf	PP.H1.abf	PP.H2.abf	PP.H3.abf	PP.H4.abf
*FCGR2A*	2.00E-316	0.88	2.47E-317	0.11	0.01
*FCGR2B*	3.02E-280	0.85	4.73E-281	0.13	0.02
*FCGR3B*	5.78E-316	0.84	7.15E-317	0.10	0.06
*IL6R*	5.92E-316	0.94	2.97E-317	0.05	0.02
*LTA*	4.74E-316	0.53	4.15E-316	0.46	0.01
*NFKB1*	2.06E-44	0.92	1.73E-45	0.08	0.01
*PLA2G1B*	6.03E-08	0.94	3.19E-09	0.05	0.01
*TNF*	3.18E-25	0.53	2.80E-25	0.47	0.01

## 4 Discussion

We leveraged *cis*-pQTL and GWAS summary statistics to estimate the effects of genetically perturbed DMARD targets on the risk of AD. To our knowledge, this is the first study to investigate DMARD targets and AD risk using a combined MR and colocalization approach.

We found limited evidence to support the repurposing potential of select DMARD targets for reduced AD risk. The MR analyses provided little evidence that eight out of the nine selected targets—*ACE2*, *FCGR2A*, *FCGR2B*, *IL6R*, *LTA*, *NFKB1*, *PLA2G1B*, and *TNF*—affected the risk of AD. Our MR results suggest that a 1 SD unit increase in plasma *FCGR3B* concentration increases AD risk by 10%. Though, further analysis of genetic signaling in the flanked region on chromosome 1 revealed that *FCGR3B* did not colocalize with AD. It is possible for MR and colocalization to provide conflicting findings. As both H3 and H4 were low, this may not necessarily indicate evidence against colocalization (Giambartolomei et al., 2018). Rather, one explanation for the colocalization findings may be a lack of power, demonstrated by a high strength of association with the exposure dataset (PP.H1 = 83.5%), but not with the IGAP dataset (PP.H2 = 7.15E-315 %) (Giambartolomei et al., 2018; Zuber et al., 2022). Thus, the discordance between our MR and colocalization results for *FCGR3B* should not be interpreted as contradicting the MR estimate, which remains indicative of a causal effect on AD risk (Zuber et al., 2022).

Of interest, a recent large-scale proteogenomic analysis of cerebrospinal fluid (CSF) identified a *cis*-association for *FCCGR3B* and AD risk through a proteome-wide association study (PWAS) and colocalization (Western et al., 2024). Their PWAS association statistic showed an opposite effect direction for *FCGR3B* than our MR estimate, which may be explained by differences in tissue-specific gene expression and subsequent downstream effects on protein levels, as gene expression varies greatly between brain and non-brain tissues (Magger et al., 2012; GTEx Consortium, 2017). The direct proximity of CSF tissue to the brain makes it one of the most relevant biomarkers for neurodegenerative disease (Karlsson et al., 2024). Plasma, in contrast, has much greater protein concentration and availability of proteomic data, rendering it a useful and accessible tool for analysis (Reiber, 2001). With both our study as well as the referenced paper having found some evidence of an association between *FCGR3B* and AD risk, future research should further investigate *FCGR3B* across tissues (e.g., plasma, CSF) to better elucidate its causal role in AD risk and possible therapeutic viability.

The evidence in our study suggests that *LTA*, *FCGR2A*, *FCGR2B*, and *PLA2G1B* are unlikely to be effective candidate targets for AD; these findings should be taken into consideration to reduce the high attrition rate in AD drug development by discouraging trials of likely irrelevant targets (Cummings et al., 2014). While our MR analysis also did not prioritize *ACE2*, it should be borne in mind that the effect estimate was obtained by instrumenting *trans*-acting pQTLs—as *ACE2* resides on chromosome X, of which the IGAP study did not have data for. Therefore, our estimate may not be as reflective of the protein effects as had we been able to instrument *cis*-acting pQTLs within the gene locus. Additionally, we observed little evidence of an effect for *IL6R.* However, our estimate shows a consistent direction of effect with that of a recent MR study reporting significant protective effects of *IL6R* variants on AD risk via downstream C-reactive protein serum levels (Myserlis et al., 2024). As that study utilized data from larger-scale GWASs, including ‘proxy’ AD cases, as opposed to the present study which only examined clinically diagnosed AD cases, it is possible that with greater power, we may have similarly detected an effect of *IL6R*. Alternatively, it is plausible that they observed an effect of *IL6R* due to bias introduced by the inclusion of ‘proxy’ AD cases. Definitive conclusions cannot be drawn regarding the efficacy of *NFKB1* and *TNF* due to the imprecision of estimates observed in this study. A previously conducted MR study found little evidence of a causal effect of *TNF* serum levels on AD (Andrews and Goate, 2019). Their instrumentation of pQTLs derived from blood serum raises the concern of tissue relevance as mentioned earlier in the discussion of *FCGR3B*. However, the strength of associations between *TNF* levels in other tissues, such as CSF, and AD risk is unclear. In a review of the literature, no MR analyses have yet been conducted to explore this, and correlational studies yield ambiguous findings regarding the association of CSF *TNF* with AD risk and symptom progression (Swardfager et al., 2010; Aljuhani, 2025). While future studies are recommended to assess the relationship between *TNF* and AD risk in a more relevant tissue for neurodegeneration such as CSF, the potential effect of plasma *TNF* merits further exploration as well.

Several limitations should be considered. First, this study was limited by the lack of proteomic data available for relevant drug targets. Among the 33 initially selected targets from 11 DMARDs, the final MR analyses could include only nine targets from eight DMARDs. Notably, methotrexate had to be excluded, despite recent observational evidence suggesting an association with reduced AD risk (Newby et al., 2020). This highlights the importance of greater funding for proteome-based genome-wide association studies. Efforts should also be directed towards integrating the X chromosome into genome-wide analyses. While the unavailability of X chromosome mapping in the IGAP dataset did not impact most of our targets, we were limited to running the MR analysis on *ACE2* (Xp22.2) via *trans*-acting SNPs located on the autosomal chromosomes, while colocalization could not be performed. This may hinder our study as angiotensin-converting enzyme (*ACE*), of which *ACE2* is its homologue, has been linked to Aβ plaque degradation and is recognized as a target for intervention in AD (Kunkle et al., 2019; Jochemsen et al., 2014). Another limitation of this study is tissue specificity of gene and protein expression (Bossi and Lehner, 2009; Whitehead and Crawford, 2005). As we only examined plasma, future studies that instrument variants across various tissues and, ideally, in tissue more relevant to neurodegeneration (e.g., CSF) are encouraged and may yield different results. Additionally, there are several caveats to drug target MR that limit the ability to causally examine broader drug usage and disease outcome associations (Anderson and Williams, 2023). As we estimated the effects of perturbing individual drug targets, we cannot infer the likely aggregate effects of specific DMARDs. Furthermore, it is difficult to fully understand the pharmacodynamics of medications and their mechanisms of action (MOA) (e.g., the exact molecular pathways by which etanercept affects *FCGR3B* and other targets). Thus, based solely on genetic evidence, we cannot conclude how prescribed etanercept use would affect AD risk via *FCGR3B* and other known or unknown targets. We therefore recommend further mechanistic studies and clinical trials that can systematically account for MOAs for a more comprehensive evaluation of DMARD use. Finally, it is worth noting possible biases which could have further attenuated our estimates. Sourcing instruments from the UK Biobank, which is prone to selective representation, may introduce collider bias (Gkatzionis and Burgess, 2019; Schoeler et al., 2023). Selected study participants tend to be healthier and may therefore have lower genetic liability to disease traits which, in turn, may affect MR estimates (Munafò et al., 2018). That said, to date, there is little evidence in the literature specifically linking proteomic data to selection bias.

## 5 Conclusion

The findings from our study do not support the majority of selected DMARD targets as efficacious treatment candidates for AD, with exception of the etanercept target, *FCGR3B*, which was prioritized by MR analysis and warrants further investigation. Our findings should be considered within the current discourse on targeting inflammatory mechanisms for AD treatment and prevention. Larger-scale MR and colocalization studies that can instrument a greater number of DMARD targets across various tissues are additionally recommended.

## CRediT authorship contribution statement

**Christina N. Kushnir:** Writing – review & editing, Writing – original draft, Visualization, Investigation, Formal analysis, Data curation, Conceptualization. **Victoria Taylor-Bateman:** Visualization, Software, Investigation. **Neil M. Davies:** Writing – review & editing, Validation, Supervision, Project administration, Methodology, Funding acquisition, Conceptualization. **Emma L. Anderson:** Writing – review & editing, Validation, Supervision, Methodology, Funding acquisition, Data curation, Conceptualization.

## Ethics and consent statement

The GWASs included in this study obtained written informed consent from all participants prior to data collection. Related study protocols were approved accordingly by the appropriate institutional review boards accordingly. Therefore, separate ethical approval was not required for the present study.

## Availability of data and materials

Proteogenomic summary data from the UKB-PPP can be found at http://ukb-ppp.gwas.eu. Full AD GWAS data can be obtained by requesting access via the National Institute on Aging Genetics of Alzheimer's Disease Data Storage Site (NIAGADS) data repository, ID: NG00075. The codes used to generate the results of this study are located at https://github.com/tkush15/AD_DMARD_MRCOLOC**.**

## Declaration of generative AI

No generative AI or AI-assisted technologies were used in the writing process.

## Funding

ELA is supported by a UKRI Future Leaders Fellowship (MR/W011581/2). NMD is supported via a Norwegian Research Council 295989.

## Declaration of competing interest

The authors declare that they have no known competing financial interests or personal relationships that could have appeared to influence the work reported in this paper.

## Data Availability

Links to the exposure and outcome data as well as to coding used during analysis can be found in the Availablity of data and materials section.
